# Feminization of Male Mouse Liver by Persistent Growth Hormone Stimulation: Activation of Sex-Biased Transcriptional Networks and Dynamic Changes in Chromatin States

**DOI:** 10.1128/MCB.00301-17

**Published:** 2017-09-12

**Authors:** Dana Lau-Corona, Alexander Suvorov, David J. Waxman

**Affiliations:** Department of Biology and Bioinformatics Program, Boston University, Boston, Massachusetts, USA

**Keywords:** DNase hypersensitivity, sexual dimorphism, hepatocellular carcinoma, cytochrome P450, liver zonation, hypophysectomy, Trim24, H3-K27me3, DHS

## Abstract

Sex-dependent pituitary growth hormone (GH) secretory profiles—pulsatile in males and persistent in females—regulate the sex-biased, STAT5-dependent expression of hundreds of genes in mouse liver, imparting sex differences in hepatic drug/lipid metabolism and disease risk. Here, we examine transcriptional and epigenetic changes induced by continuous GH infusion (cGH) in male mice, which rapidly feminizes the temporal profile of liver STAT5 activity. cGH repressed 86% of male-biased genes and induced 68% of female-biased genes within 4 days; however, several highly female-specific genes showed weak or no feminization, even after 14 days of cGH treatment. Female-biased genes already in an active chromatin state in male liver generally showed early cGH responses; genes in an inactive chromatin state often responded late. Early cGH-responsive genes included those encoding two GH/STAT5-regulated transcriptional repressors: male-biased BCL6, which was repressed, and female-specific CUX2, which was induced. Male-biased genes activated by STAT5 and/or repressed by CUX2 were enriched for early cGH repression. Female-biased BCL6 targets were enriched for early cGH derepression. Changes in sex-specific chromatin accessibility and histone modifications accompanied these cGH-induced sex-biased gene expression changes. Thus, the temporal, sex-biased gene responses to persistent GH stimulation are dictated by GH/STAT5-regulated transcription factors arranged in a hierarchical network and by the dynamics of changes in sex-biased epigenetic states.

## INTRODUCTION

Sexual dimorphism is a characteristic of liver physiology and pathology (1). Males and females exhibit differences in hepatic drug and lipid metabolism, which impact cholesterol sensing and transport (2), as well as pharmacokinetics and pharmacodynamics linked to sex differences in drug efficacy, drug toxicity, and liver disease susceptibility (3, 4). Females are more susceptible to acute liver failure, autoimmune hepatitis, and toxin-mediated hepatotoxicity (5), while males are more susceptible to liver fibrosis progression (6) and hepatocellular carcinoma (7–9). Underlying these phenotypic sex differences are sex differences in the expression of an unexpectedly large number of genes, as shown in mouse, rat, and human livers (10–13), including genes involved in lipid metabolism associated with dyslipidemia and cardiovascular disease risk in humans (13).

Growth hormone (GH), acting through its sex-specific plasma profiles and cell surface receptor (14), is the major hormonal determinant of sex differences in adult liver. GH secretion by the anterior pituitary gland is controlled by the hypothalamus and regulated by metabolic cues (15) and shows sexual dimorphism in many species, including rodents and humans (16–18). In the rat and mouse, pituitary GH secretion is intermittent in males, whereas in females, GH is secreted more frequently, and consequently, target tissues are stimulated by GH in a nearly continuous (persistent) manner (19, 20). GH secretion patterns are set by gonadal steroids during the neonatal period, are first manifested at puberty, and continue into adulthood (15, 21). Ablation of circulating GH by hypophysectomy abolishes ∼90% of liver sex differences, and exogenous GH given either in pulses (male pattern) or continuously (female-like pattern) restores the corresponding sex-biased patterns of liver gene expression (22–24).

The transcription factor STAT5b is a major essential mediator of the sex-dependent transcriptional responses of the liver to GH, as seen in mouse knockout models (10, 25). Liver STAT5 activity directly correlates with the occurrence of a plasma GH pulse in male rats (26, 27), whereas in female rats liver STAT5 activity persists due to the more persistent stimulation of hepatocytes by GH (28). Comparison of the sex-differential, GH-induced liver STAT5 activation patterns between rats (26) and mice (29) indicates that the key sex difference is the occurrence of a sustained period of time when STAT5 is inactive in male liver. The sex-specific transcriptional actions of GH-activated STAT5 are enhanced by several GH/STAT5-regulated transcription factors, including BCL6 (30), a male-biased transcriptional repressor (31) that preferentially represses female-biased genes in male liver (29), and CUX2 (32), a female-specific repressor (33) that downregulates a subset of male-biased genes in female liver but can also activate a subset of female-biased genes (34).

Information on chromatin accessibility, histone modification, and transcription factor binding can be integrated to elucidate complex patterns of transcriptional regulation. Genomic sites hypersensitive to cleavage by DNase I (DNase hypersensitive sites [DHS]) correspond to accessible (open) chromatin regions, which encompass key regulatory elements, including enhancers, promoters, insulators, and silencers, and are often flanked by specific histone modifications (35). Genome-wide DHS mapping in mouse liver has established a close relationship between sex differences in chromatin accessibility and sex differences in gene expression (36, 37). Sex-biased STAT5 binding to liver chromatin is strongly enriched at sex-biased DHS and correlates positively with sex-biased activating histone marks and negatively with repressive marks (29, 37). Differences in the epigenetic regulatory mechanisms utilized in male and female livers are apparent, with many of the most highly female-biased genes specifically subject to H3-K27me3-based repression in male liver (37).

GH regulation of sex-biased gene expression thus involves complex interactions between transcriptional networks, genomic regulatory elements, and epigenetic features. Fundamental questions remain regarding the mechanisms by which GH establishes and maintains the sex-biased chromatin states that determine the sex-biased expression of many GH target genes (37). Continuous GH (cGH) infusion of male mice overrides the endogenous male, pulsatile GH pattern, thereby abolishing the male-specific, pulsatile pattern of liver STAT5 activity (29). cGH infusion also closes many male-biased DHS and opens female-biased DHS in male liver (36), while downregulating several male-biased genes and upregulating female-biased genes (24). We utilize here the cGH infusion model to carry out a global gene expression time course study to elucidate the transcriptional events that lead to feminization of male mouse liver. Our findings reveal that cGH infusion induces distinct, time-dependent waves of male-biased gene repression and female-biased gene derepression associated with changes in the sex-biased liver chromatin environment and initiated by a hierarchical transcriptional network comprised of several sex-biased transcription factors.

## RESULTS

### Continuous GH-responsive sex-biased genes in mouse liver.

**FIG 1:**
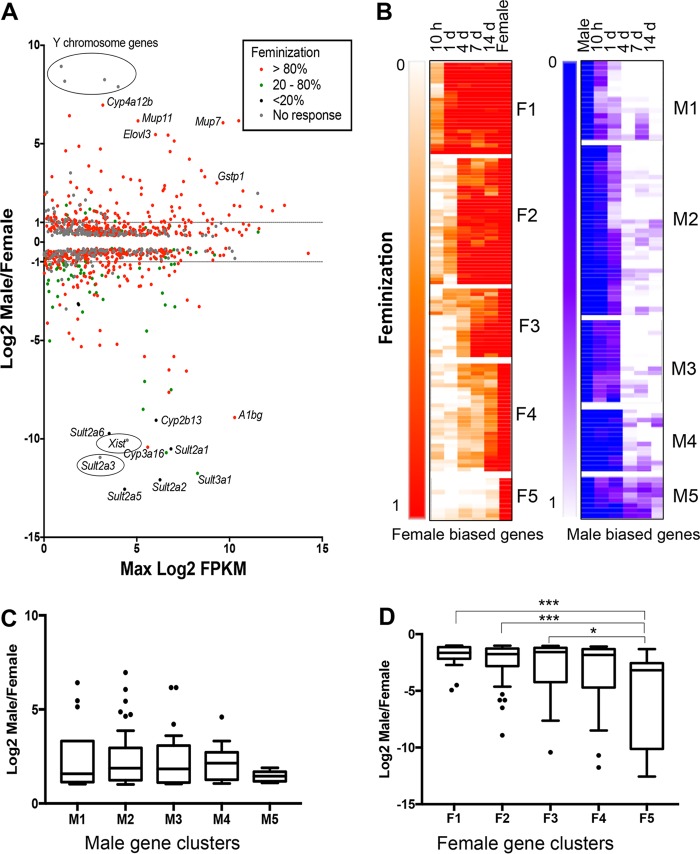
cGH responsive genes in male liver. (A) MA plot showing the log_2_ sex ratio compared to gene expression level, in log_2_ FPKM units, for 983 sex-biased genes (EdgeR *P*_adj_ < 0.01, FPKM > 1). Genes are colored based on the maximum percent feminization after cGH infusion. Data points are identified for select genes. (B) Hierarchical clustering was applied to 213 cGH-responsive sex-biased genes based on the feminization values at each time point. Heat maps show findings for 120 female-biased genes (≥2-fold sex bias) in five clusters (F1 to F5) and 93 male-biased genes (≥2-fold sex bias) in five clusters (M1 to M5). For female-biased genes the female gene feminization level is set to 1, and for male-biased genes the male gene feminization level is set to 0. (C and D) Gene expression log_2_ sex ratios for cGH-responsive male-biased (C) and female-biased (D) genes for each of the cGH response clusters. Significance values by ANOVA are indicated as follows: *, *P* < 0.05; **, *P* < 0.01; and ***, *P* < 0.001.

Adult male mice were treated with GH given as a continuous infusion (cGH) for times ranging from 10 h to 14 days to override the male pulsatile GH secretion pattern. Transcriptome sequencing (RNA-seq) analysis of liver mRNA identified 983 liver-expressed, sex-biased genes (see Table S1 in the supplemental material), of which 74% showed feminized expression, i.e., suppression of male-biased genes and induction of female-biased genes, following cGH infusion. Moreover, 69% of the cGH-responsive genes were fully feminized at their maximal response time point (Fig. 1A). Of the 255 sex-biased genes with a sex ratio >2 (142 female-biased genes and 113 male-biased genes, excluding four Y-chromosome genes), 233 (91%) were cGH responsive (see Tables S2 and S3 in the supplemental material). The majority of these stringent sex-biased genes responded within 4 days (86% of male-biased genes and 68% of female-biased genes with a feminization of ≥20%). In contrast, only 62 (0.9%) of 7,225 stringently sex-independent genes responded to cGH infusion at any time point (see Table S4 in the supplemental material).

**FIG 2:**
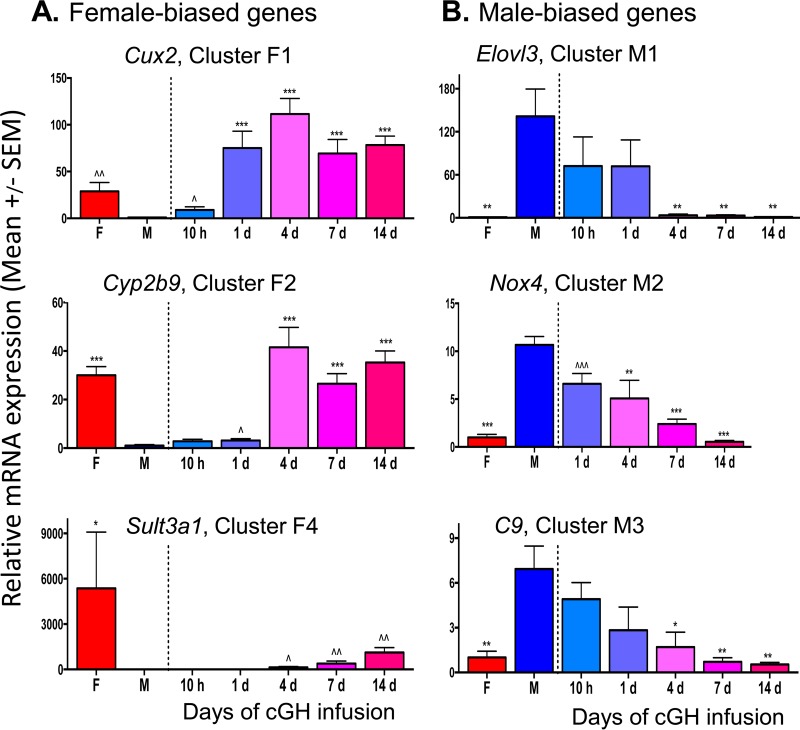
qPCR expression of cGH responsive sex-biased genes. RT-qPCR analysis of total liver RNA was performed using gene-specific mRNA primers for the indicated sex-biased genes in gene clusters F1, F2, and F4 (A) and in gene clusters M1, M2, and M3 (B). The data shown are means ± the SEM based on *n* = 6 to 10 livers per group. Significance values by ANOVA are indicated as follows: *, *P* < 0.05; **, *P* < 0.01; and ***, *P* < 0.001. The symbol “^” indicates that statistical significance was reached only by *t* test and not ANOVA (^, *P* < 0.05; ^^, *P* < 0.01; and ^^^, *P* < 0.001). Primers used for qPCR analysis are shown in Table S6 in the supplemental material.

Hierarchical clustering of a set of 213 sex-biased genes selected for consistent patterns of GH responsiveness using STEM (38) (see Materials and Methods) identified five clusters of female-biased genes (F1 to F5), and five other clusters of male-biased genes (M1 to M5) (Fig. 1B and Table 1). cGH response patterns were validated for select genes by quantitative reverse transcription-PCR (RT-qPCR). *Cux2*, a cluster F1 female-biased gene, was significantly induced after 10-h cGH infusion and was fully feminized (≥100% intact female expression level) within 1 day; in contrast, *Cyp2b9*, in cluster F2, was not significantly induced by cGH until day 4 (Fig. 2A). Several highly female-biased *Sult* and *Cyp* genes in clusters F4 and F5 (Fig. 1A and Table 1) were only partially feminized after 14 days of cGH treatment, as shown for S*ult3a1* (Fig. 2A). *Elovl3*, a male-biased gene in cluster M1, reached >90% feminization by 4 days of cGH infusion (Fig. 2B). Similarly, *Nox4*, in cluster M2, was significantly downregulated by 1 day of cGH but required longer time for full feminization. *C9*, in cluster M3, showed partial responses at the early time points and was completely feminized (i.e., repressed) after 4 days. Genes in cluster M4 showed no response at the early time points but were completely feminized by 4 days of cGH infusion or later (Fig. 1B). Genes in cluster M5 were only partially repressed after 14 days of cGH infusion (Table 1). KEGG pathway analysis of the genes in each cluster revealed enrichment for metabolism gene pathways in all of the clusters (see Table S7 in the supplemental material).

**TABLE 1:** Feminization of cGH-responsive sex-biased gene clusters displayed in Fig. 1B

Cluster	Mean feminization of cluster (%)					No. of genes	Examples
	10 h	1 day	4 days	7 days	14 days		
Male-biased genes							
M1	61	88	>100	>100	>100	15	*Cyp7bI*, *Elovl3*, *Mup16*
M2	6	60	>100	99	>100	39	*Nox4*, *Ugt2b38*, *Cyp4a12*
M3	40	41	>100	>100	>100	19	*Mup20*, *Gstp1*, *Selenbp2*
M4	0	2	92	89	>100	13	*Mup14*, *Mup17*, *Alas2*
M5	0	0	65	60	79	7	*Serpina1d*, *Slc17a8*, *Dpy1913*
Not responsive						10	*Mir293*, *Gm4841*, *Gm4956*
Total						103	
Female-biased genes							
F1	39	>100	>100	>100	>100	26	*Trim24*, *Sall1*, *Cux2*
F2	13	22	>100	>100	>100	37	*Cyp2a4*, *Cyp2b9*, *Pnpla3*
F3	8	14	35	82	>100	18	*Cyp3a16*, *Ugt2b37*, *Acot2*
F4	8	3	26	30	64	26	*Fmo3*, *Sult3a1*, *Tox*
F5	4	1	5	4	16	13	*Sult2a1*, *Sult2a5*, *Cyp2b13*
Not responsive						12	*Ankrd55*, *Raet1e*, *Xist*
Total						132	

### Early transcriptional changes indicated by intronic sequence reads.

**FIG 3:**
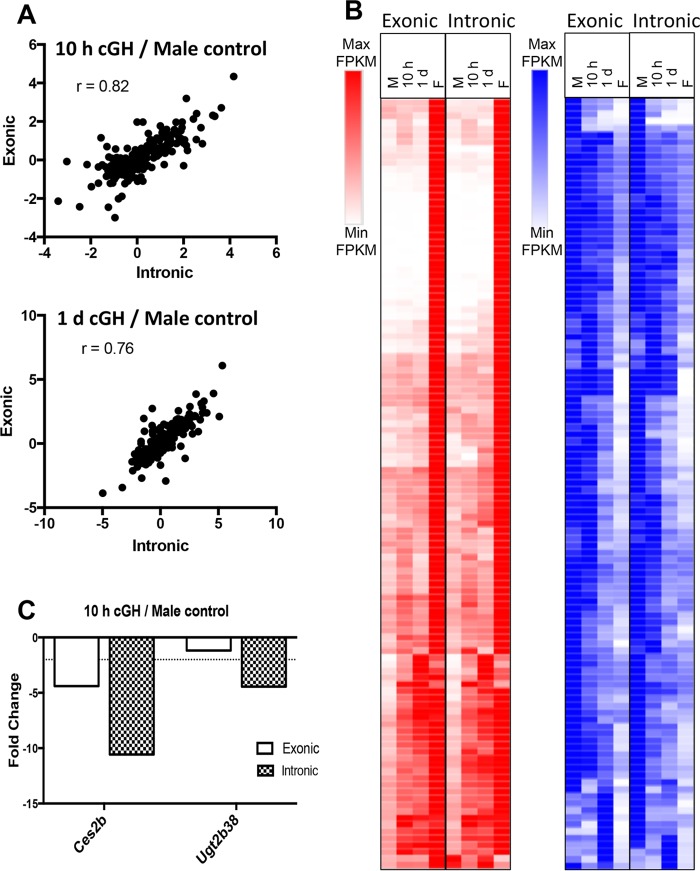
Analysis of intronic reads from rRNA-depleted RNA-seq. (A) Pearson correlation of exonic read to intronic read ratios after 10 h and 1 day of cGH infusion. Analysis is based on the set of 226 genes with a >2-fold sex bias and having sufficient intronic reads per transcript for reliable quantification. (B) Heat map of the relative expression values at each time point comparing intronic and exonic read counts, scaled based on the maximum FPKM value for each gene. (C) *Ces2b* and *Ugt2b38* show a >2-fold difference in fold change in primary transcript levels based on the intronic and exonic read counts after 10 h of cGH treatment.

Since RNA abundance measured by RNA-seq is a function of both RNA synthesis and stability, differences in the stability of individual male-biased liver mRNAs could impact the time required for those mRNAs to decline following cGH infusion. Intronic sequence reads assayed by RNA-seq primarily correspond to unprocessed transcripts whose abundance reflects the nascent transcription rate; they can therefore be compared to exonic sequence reads to distinguish transcriptional from posttranscriptional regulation (39, 40). Accordingly, we quantified cGH-induced gene expression changes separately for exonic reads and intronic reads extracted from our poly(A)-liver RNA-seq data set (see Materials and Methods). Similar overall cGH response patterns were apparent when comparing exonic and intronic reads (see Fig. S1 in the supplemental material), consistent with the effects of cGH largely being transcriptional. As anticipated, more rapid decline in expression was apparent for some of the male-biased genes when monitored using intronic reads. To better assess changes in nascent transcription rates, we obtained rRNA-depleted RNA-seq data for male livers treated with GH for 10 h or 1 day and from sham-treated male and control female livers, removing the bias toward spliced RNA that poly(A) selection can introduce (see Table S8 in the supplemental material). The cGH-induced changes in expression were strongly correlated between exonic and intronic read data at both time points (*r* = 0.82 at 10 h cGH; *r* = 0.76 at 1 day of cGH) (Fig. 3A). Furthermore, the overall response patterns were very similar (Fig. 3B). Only two male-biased genes, *Ces2b* and *Ugt2b38*, showed a significant, >2-fold difference in response between intronic and exonic reads after 10 h of cGH treatment (Fig. 3C). These two male-biased genes are direct targets of STAT5, and their transcription is rapidly induced by each successive GH pulse in male liver (41). Further, the primary transcript levels (i.e., hnRNA) of these two genes peak earlier than mature RNA levels when a GH pulse is given to hypophysectomized male mice (41). Accordingly, these male-biased genes are particularly sensitive to the loss of pulsatile STAT5 activity secondary to cGH infusion (29) and, consequently, their primary transcripts, measured by intronic read counts, are expected to show the earlier downregulation compared to the mature RNAs (exonic sequence reads) seen in Fig. 3C. Two other STAT5 target genes that are also induced by GH pulse-activated STAT5, *Igf1* and *Cish* (41, 42), were not repressed by cGH infusion. *Igf1* and *Cish* can apparently be induced by either pulsatile or persistent STAT5 stimulation, consistent with their being expressed at similar levels in male and female livers (41).

### Time-dependent regulation by cGH-responsive transcription factors.

**FIG 4:**
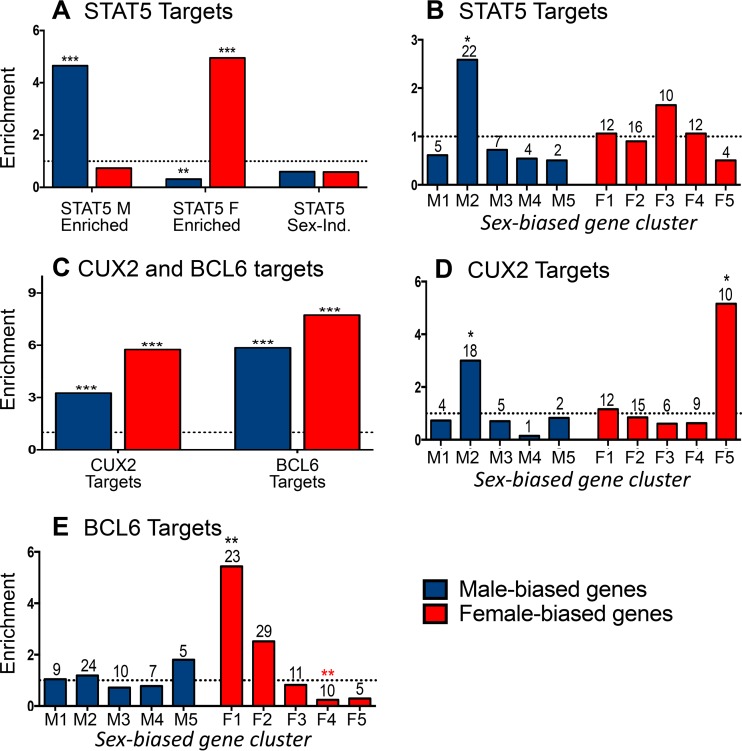
Regulation by sex-biased transcription factors. (A) Male-enriched (1,765 sites), female-enriched (1,790 sites), and sex-independent (11,531 sites) STAT5 binding sites were mapped to their target genes using GREAT. Enrichment for male-biased (blue) and female-biased (red) gene targets was computed compared to a background set of 7,225 stringent sex-independent genes. (B) Enrichment of the male-biased target genes of male-enriched STAT5 binding sites in each of the cGH-responsive male gene clusters (M1 to M5) and enrichment of female-biased target genes of the female-enriched STAT5 binding sites in each of the cGH-responsive female gene clusters (F1 to F5). (C) CUX2 and BCL6 binding sites (1,471 and 6,432, respectively) were mapped to their target genes, and enrichment for both male-biased and female-biased target genes was computed compared to a background set of stringent sex-independent genes. (D) CUX2 female-biased and male-biased gene target enrichment in each cGH response clusters as described for panel B for STAT5. (E) BCL6 female-biased and male-biased gene target enrichment in each cGH response cluster, as described in panel B. The enrichment of STAT5, CUX2, and BCL6 targets for being enriched in each of the gene sets examined was calculated as described in Materials and Methods. Significant enrichment or depletion by the Fisher exact test is indicated by black or red asterisks, respectively (*, *P* < 0.05; **, *P* < 0.01; and ***, *P* < 0.001). Numbers above the bars indicate the number of gene targets in each gene set. Similar results were obtained for STAT5 and BCL6 target enrichment when the target genes of each factor were defined as the nearest gene within 10 kb (data not shown).

Next, we examined the impact of cGH infusion on all sex-biased genes that are direct targets of STAT5 to obtain a global view of the impact of the change from pulsatile to persistent liver STAT5 activity with cGH treatment. Liver STAT5 binding sites identified by chromatin immunoprecipitation and sequencing (ChIP-seq), including binding sites significantly stronger in males or in females (male-enriched and female-enriched STAT5 binding sites, respectively) (29), were mapped to their putative target genes using GREAT (43) (see Materials and Methods). Sex-biased STAT5 binding was observed nearby 44% of all sex-biased genes, with gene targets of male-enriched STAT5 binding significantly enriched for male-biased genes (enrichment score [ES] = 4.6, *P* = 2.98E−12) and gene targets of female-enriched STAT5 binding significantly enriched for female-biased genes (ES = 4.9, *P* = 2.2E−16) (Fig. 4A), consistent with our earlier findings (29). Further, 100% of the male-biased gene targets of male-enriched STAT5 binding sites were repressed by cGH infusion, and 96% of the female-biased gene targets of female-enriched STAT5 binding sites were induced by cGH infusion. Given these significant responses, we investigated whether the sex-biased STAT5 target genes show a preferential distribution across the defined clusters of time responsiveness to cGH infusion (Fig. 1B). Male-biased genes targeted by male-biased STAT5 binding sites were enriched in gene cluster M2 (ES = 2.58, *P* = 6.06E−4) (Fig. 4B, left), whose genes are significantly repressed 1 day after liver STAT5 pulsation is abolished by cGH infusion. Of note, 81% (18/22) of the male-biased STAT5 targets in cluster M2 are repressed in male liver following hypophysectomy (type I gene response [41]), and 68% of these genes are downregulated in STAT5b-deficient male liver, as determined by microarray analysis (10), indicating these genes are positively regulated by GH pulse-activated STAT5. Conversely, the genes targeted by female-biased STAT5 binding sites did not show significant preferential enrichment in any of the cGH response clusters (Fig. 4B, right).

Two other sex-biased, GH-regulated transcription factors work together with STAT5 to enforce the sex-specific effects of GH in the liver. BCL6, a GH/STAT5-regulated, male-biased transcriptional repressor, preferentially binds to and represses female-biased genes in male liver, in part by competing for STAT5 binding (29); and CUX2, a GH/STAT5-regulated female-specific repressor, both represses a subset of male-biased genes and activates a subset of female-biased genes in female liver (34). At the earliest cGH time point (10 h), we observed (i) significant induction of *Cux2*, as well as *Trim24*, a female-biased CUX2 target gene that may in part mediate CUX2's downstream effects (34), and (ii) significant downregulation of *Bcl6* (Table 2). A third female-biased transcription factor gene, *Sall1*, which is also a target of CUX2 binding (34), was also induced at this time (Table 2).

**TABLE 2:** Sex biased transcriptional regulators^*a*^

Gene	Sex specificity	M/F ratio	Feminization at 1 day (%)	Onset of sex specificity (wks)	Response to:	
					Cux2 siRNA	Adeno Cux2
*Bcl6*	Male	1.9	94	4	Up	Down
*Sall1*	Female	−2.9	>100	4	Down	No change
*Trim24*	Female	−3.3	>100	4	Down	Up
*Cux2*	Female	−30.3	>100	4	Down	Up

a The findings for four sex-biased transcriptional regulators that were significantly induced or repressed after 1 day of cGH infusion are presented. The onset of sex specificity is according to reference 54, and the response to Cux2 knockdown or overexpression is according to reference 34.

We hypothesize that the cGH-induced changes in expression seen for many sex-biased genes are secondary to the induction or repression of these early GH-responsive sex-biased transcriptional regulators. To test this hypothesis, we investigated the cGH time-responsiveness of the gene targets of CUX2 (34) and BCL6 (29), as follows. A total of 1,471 CUX2 binding sites identified in female liver (34) were mapped to their target genes, which were significantly enriched for both male-biased and female-biased genes (ES = 3.2, *P* = 2.19E−07 and ES = 5.7, *P* < 2.2E−16, respectively) compared to sex-independent genes (Fig. 4C). Importantly, the male-biased CUX2 target genes were enriched in the gene set showing early repression by cGH (cluster M2; ES = 3.0, *P* = 0.02; Fig. 3D). Further, we determined that 65% of the male-biased CUX2 targets repressed by cGH are also repressed when *Cux2* is overexpressed (34) in male liver (see Table S2 in the supplemental material). Thus, the early induction of *Cux2* by cGH infusion is associated with the direct (34) and rapid repression of male-biased genes. CUX2 gene targets are also enriched in the subset of late cGH-responding female-biased genes (cluster F5; ES = 5.15, *P* = 0.04), and 50% of these genes are induced in male liver by *Cux2* overexpression (see Table S3 in the supplemental material).

To investigate the effects of cGH on BCL6 targets, 6,432 BCL6 binding sites previously identified in male liver (29) were mapped to gene targets. These BCL6 target genes showed strong enrichment for both male-biased and female-biased genes (ES = 5.8, *P* < 2.2E−16, and ES = 7.7, < 2.2E−16, respectively) (Fig. 4C). Importantly, the female-biased BCL6 target genes are strongly enriched for early cGH-inducible genes (cluster F1; ES = 5.43, *P* = 0.004) and are depleted of late cGH-inducible genes (cluster F4; ES = 0.23, *P* = 0.0022) (Fig. 4E), consistent with our earlier finding that BCL6 preferentially represses female-biased genes in male liver (29). Together, these findings indicate that the early induction of *Cux2* and the early repression of *Bcl6* by cGH infusion both contribute to the subsequent repression of male-biased genes and induction of female-biased genes in male liver.

### Continuous GH-induced epigenetic changes at sex-biased genes.

**FIG 5:**
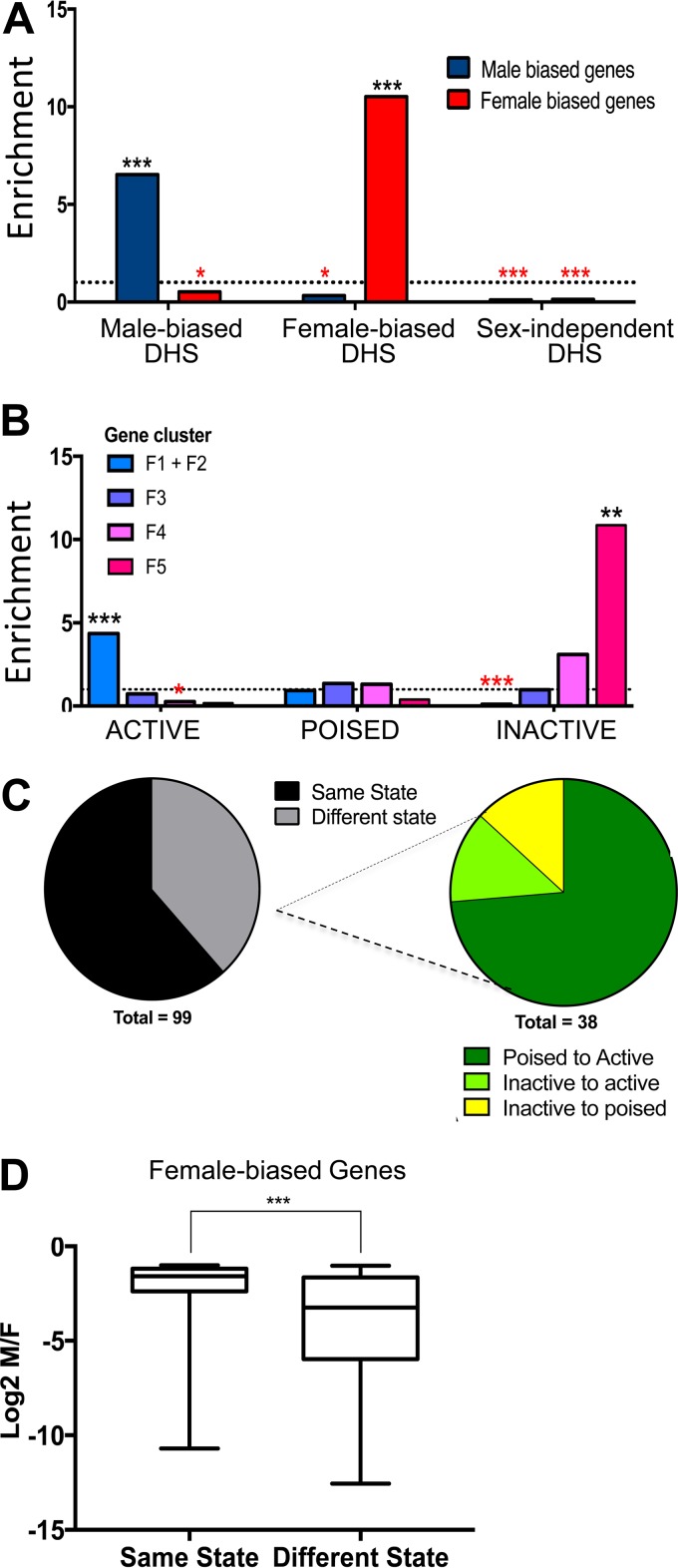
Epigenetic regulation of cGH-responsive sex-biased genes. (A) Male-biased (*n* = 2,800), female-biased (*n* = 1,379), and sex-independent liver DHS (*n* = 68,682) were mapped to their target genes using GREAT. DHS target enrichment or depletion (below dashed line) for male-biased and female-biased genes was calculated compared to a background set of stringent sex-independent genes. (B) Female-biased genes grouped by their chromatin state in male liver as described in the text. The enrichment or depletion of genes in active, poised, or inactive chromatin states (as defined previously [37]) for being early (clusters F1 and F2), intermediate (cluster F3), or late responding to cGH (clusters F4 and F5) was tested compared to all cGH-responsive female-biased genes. Significant enrichment or depletion by the Fisher exact test is indicated by black or red asterisks, respectively (*, *P* < 0.05; **, *P* < 0.01; ***, *P* < 0.001). (C, left) Proportion of female-biased genes that are in a different local chromatin state in male compared to female liver. (Right) Chromatin state transitions from male to female liver for the 38 genes in a different chromatin state between the sexes. (D) Genes that are in a different chromatin state in male versus female liver are in a more active state in female liver and show greater female specificity, as shown in the boxplot of the distribution of female specificity (log_2_ M/F expression) for female-biased genes in the same chromatin state in male and female liver (61 genes) and those in a different chromatin state (38 genes). Significance by two-tailed *t* test: ***, *P* < 0.001.

Genome-wide mapping of open chromatin sites (DHS) has shown that sex differences in gene expression are closely associated with sex differences in chromatin accessibility in adult mouse liver (36, 37). Further, cGH infusion closes 82% of male-biased DHS and open 26% of female-biased DHS after 7 days (36). Liver DHS that are male biased (*n* = 2,800), female biased (*n* = 1,379), and sex independent (*n* = 68,682) were mapped to their target genes using GREAT, and their time responsiveness to cGH was examined. Male-biased DHS gene targets were significantly enriched in male-biased genes (ES = 6.5, *P* < 2.2E−16) and female-biased DHS gene targets were significantly enriched in female-biased genes (ES = 10.5, *P* < 2.2E−16) (Fig. 5A), consistent with our earlier findings (36, 37). Further, 100% of the male-biased gene targets of the male-biased DHS were repressed by cGH infusion and 97% of the female-biased gene targets of female-biased DHS were induced by cGH. However, the sex-biased DHS target genes did not show enrichment favoring any particular cGH response cluster (data not shown).

Next, we examined whether the basal chromatin state (local chromatin environment at the transcription start site [TSS] and transcription end site [TES]) in male liver impacts the time responsiveness to cGH infusion. These analyses used chromatin state designations that we previously developed for mouse liver based on DHS activity and a panel of six chromatin marks, enabling us to classify individual liver-expressed genes as being in an active, poised, or inactive chromatin state (37). High levels of DHS and activating histone marks (H3-K27ac and H3-K4me1) characterize genes in the active chromatin state, genes in the poised state have both activating marks (H3-K4me1), and repressive marks (H3-K27me3) but are missing enhancer marks (H3-K27ac), and genes in the inactive state have high read densities of the repressive mark H3-K27me3. Utilizing these designations, we found that cGH-responsive female-biased genes already in an active chromatin state in male liver are enriched for early cGH responses (clusters F1+F2; ES = 4.3, *P* = 9.76E−04) and depleted from the set of late response genes (cluster F4; ES = 0.26, *P* = 0.02). In contrast, genes in an inactive chromatin state in male liver are strongly enriched for late cGH responses (cluster F5; ES = 10.85, *P* = 0.001) and depleted from the early gene response set (ES = 0.10, *P* = 0.00011) (Fig. 5B). Thus, female-biased genes already in an active chromatin state in male liver are more rapidly induced by cGH treatment. No corresponding chromatin state enrichments were seen for the male-biased gene clusters (data not shown). Overall, 38 (38%) of the 99 female-biased genes considered here are in a more active state in female than male liver, most commonly in a poised state in male liver and in an active state in female liver (Fig. 5C), and likely need to change to the more active chromatin state for cGH to induce their expression in male liver. The sex differential chromatin state of these 38 genes is associated with a greater sex bias in expression compared to the other 61 female-biased genes, whose local chromatin state is the same in both sexes (Fig. 5D).

### Changes in DHS and K3-K27me3 at highly female-biased genes.

**FIG 6:**
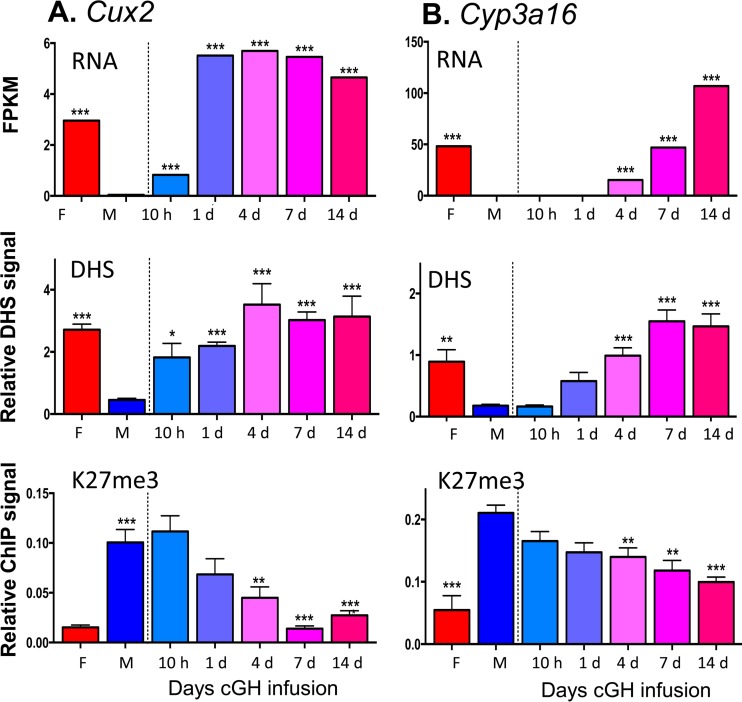
Effects of cGH infusion on expression, chromatin accessibility, and H3-K27me3 marks for the female-biased genes *Cux2* (A) and *Cyp3a16* (B). Shown at the top are RNA-seq analysis results for liver total RNA isolated from intact female and sham-treated males and from male mice infused with cGH for 10 h or for 1, 4, 7, or 14 days. Gene expression data are expressed as FPKM. Shown in the middle are the results of a qPCR analysis of genomic DNA fragments released from DNase I-digested liver nuclei isolated from individual mice from the same treatment groups shown in the top panel. qPCR was carried out using primers that target a female-biased open chromatin region nearby *Cux2* or *Cyp3a16*. DHS qPCR data were normalized to a sex-independent DHS site nearby *Tram2*. Shown at the bottom are the results of a qPCR analysis of genomic DNA fragments immunoprecipitated with anti-H3K37me3 from individual mice from the same treatment groups shown in the top and middle panels. Data were normalized to a H3K27me3-enriched site at the *Hoxb* gene region. Data shown are means ± SEM based on *n* = 6 to 10 livers per group. Significance values determined by ANOVA are indicated in each figure (*, *P* < 0.05; **, *P* < 0.01; ***, *P* < 0.001). Primers used for qPCR analysis are listed in Table S6 in the supplemental material.

**FIG 7:**
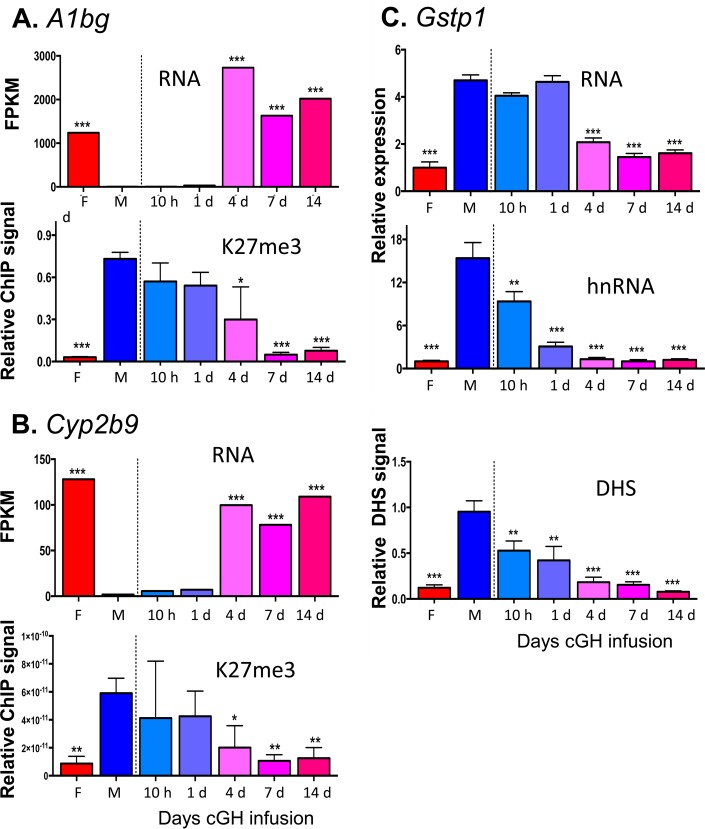
Effects of cGH infusion on expression and chromatin accessibility or H3-K27me3 marks, as indicated, for the female-biased genes *A1bg* (A) and *Cyp2b9* (B) and for the male-biased gene *Gstp1* (C). Gene expression, H3-K27me3, and DHS data were obtained for the same samples as described for Fig. 6. Panel C also compares *Gstp1* expression measured by RT-qPCR analysis using primers designed to specifically amplify either mature, spliced mRNA (top) or the primary unspliced transcript (middle). DHS data normalization and significance analysis are as described for Fig. 6. Primers used for qPCR analysis are presented in Table S6 in the supplemental material.

To investigate whether sex-biased genes, do, in fact, undergo a change in chromatin state following cGH infusion, we performed DHS analysis to identify accessible chromatin regions, as well as ChIP, for H3-K27me3, a major sex-biased repressive mark that is found across gene bodies and flanking sequences of many highly female-biased genes in male liver but not at highly male-biased genes in female liver (37). We used qPCR to assess the cGH time responsiveness of these epigenetic marks and their correlation to the transcriptional response for select sex-biased genes. *Cux2*, a highly female-biased gene in cluster F1, is significantly induced after 10 h of cGH infusion and is fully feminized after 1 day (Fig. 6A). Chromatin accessibility at a DHS in intron 2 of *Cux2* increased parallel to the induction of gene expression. The male-biased pattern of H3-K27me3 repressive marks along the *Cux2* gene body declined with cGH treatment but was delayed compared to the induction of Cux2 gene expression (Fig. 6A). Induction of *Cyp3a16*, a highly female-biased gene in cluster F3, was significantly delayed compared to Cux2, first reaching full female levels after 7 days of cGH infusion (Fig. 6B). Chromatin accessibility at a DHS 47 kb downstream of *Cyp3a16* increased parallel to the increases in gene expression. In contrast, there was a significant, but partial, decrease of H3-K27me3 along the *Cyp3a16* gene body beginning after 4 days of cGH infusion (Fig. 6B). Two other female-biased genes, *A1bg* and *Cyp2b9*, showed a cGH-induced decline in H3-K27me3 marks that coincided with the observed derepression of gene expression (Fig. 7A and B). Of note, *A1bg* and *Cyp2b9* show larger sex differences in H3-K27me3 abundance across the gene body than *Cux2* and *Cyp3a16* (see Fig. S2 in the supplemental material). These results are consistent with the expected changes in chromatin state following cGH infusion: *Cyp2b9* is already in the poised state in male liver, such that loss of H3-K27me3-mediated repression may be sufficient for gene induction in cGH-treated male liver, whereas *Cyp3a16* is in the inactive state in male liver and may require acquisition of activating marks in addition to loss of repressive marks for full feminization. Finally, *Gstp1*, a male-biased gene in cluster M3, showed a significant decrease in mRNA levels after a 4-day GH infusion (Fig. 7C). To better assess the impact of cGH infusion on *Gstp1* transcription, we quantified the levels of *Gstp1* hnRNA using primers that span an exon-intron junction. *Gstp1* hnRNA levels decreased significantly after 10 h of GH infusion, reaching the lower level of control female liver after 4 days of cGH treatment. Chromatin accessibility at a DHS 13 kb downstream of *Gstp1* decreased significantly after 10 h of cGH infusion, coinciding with the apparent changes in *Gstp1* transcription rate (Fig. 7C).

### cGH responsiveness of liver zonated sex-biased genes.

**FIG 8:**
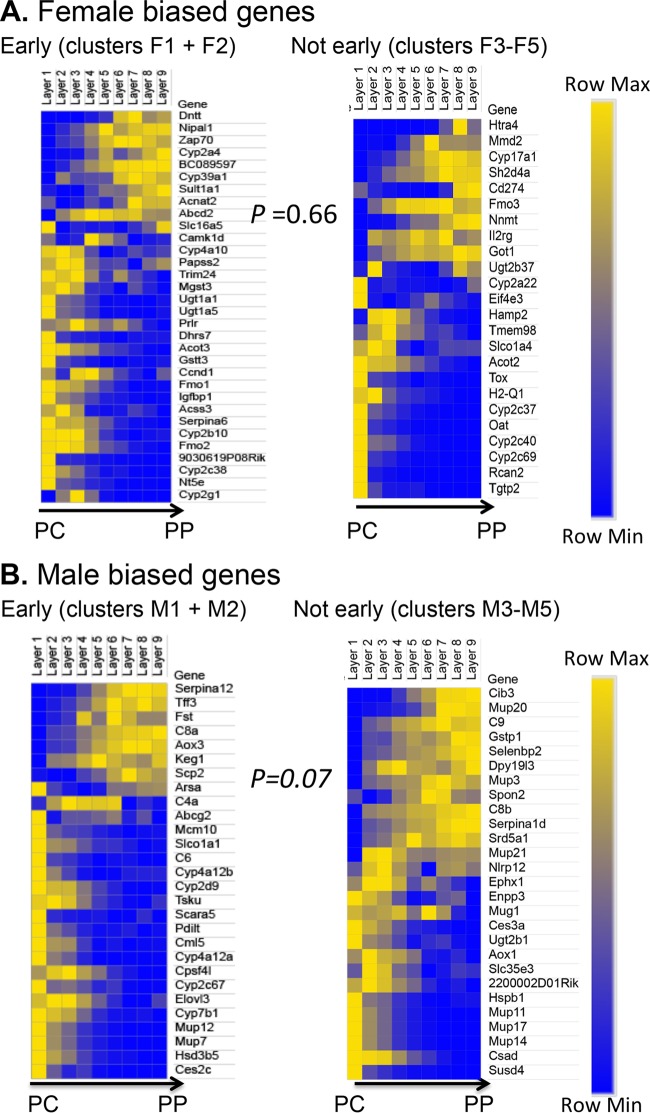
Liver zonation and response to cGH infusion. Heat maps of the relative gene expression values in each liver zone, ordered from the central vein (PC, pericentral) to the portal tract (PP, periportal), are shown, as described previously (48). Separate heat maps are shown for cGH responsive male-biased and female-biased genes that show significant spatial zonation (*q* value < 0.2), as defined earlier (48). (A) Female-biased genes, early responding (clusters F1 and F2) compared to not early responding (clusters F3 to F5). (B) Male-biased genes, early responding (clusters M1 and M2) compared to not early responding (clusters M3 to M5). Yellow, high expression; blue, low expression. Genes are ordered by the PC/PP ratio (average of zonation layers 1 to 3/average of layers 7 to 9) (48). Statistical significance was determined by the Wilcoxon rank sum test of the zonation bias of all genes in the group, as explained in Materials and Methods.

The liver shows a zonated pattern of gene expression, which imparts to hepatocytes near the portal vein (periportal cells) differences in metabolic and other functions compared to hepatocytes near the central vein (pericentral cells) (44, 45). Genes showing a zonated pattern of expression in the liver (46) might therefore have distinct sensitivities to a change in circulating GH patterns. We used genome-wide zonation profile data for male mouse liver (48) to investigate whether differences in hormonal environment between periportal and pericentral hepatocytes contribute to the differential time course of cGH responsiveness and/or to the partial responsiveness to cGH-induced feminization that characterizes some sex-biased genes. We examined zonation profiles for 229 sex-biased genes, 54% of which showed significant zonation in their expression (*q* value < 0.2); this proportion is comparable to the ∼50% of all liver-expressed genes that show nonrandom zonation (48). The rapid GH pulse-responsive (but sex-independent) genes *Igf1* and *Cish* showed significant periportal bias in expression. In contrast, some of the female-biased genes that were partially feminized by cGH infusion (clusters F4 and F5) showed strong pericentral bias (*Cyp2c40*, *Tgtp2*, and *Cyp2c37*). To investigate global patterns of zonation, we compared the zonation bias of significantly zonated sex-biased genes (125 genes) (Fig. 8) to that of significantly zonated stringent sex-independent genes (2,678 genes). No significant difference in zonation was seen between the two gene sets (*P* = 0.16 [Wilcoxon rank sum]). Furthermore, for sex-biased genes, there was no significant difference in zonation bias between early cGH response genes (clusters 1 and 2) and genes responding to cGH later (clusters 3 to 5) (*P* = 0.66 for female-biased and *P* = 0.07 for male-biased genes [Wilcoxon rank sum]) (Fig. 8). A caveat of this analysis is that many of the most highly female-biased genes show little or no expression in the male liver data set used to define liver zonation (48) and were therefore necessarily excluded from this analysis.

## DISCUSSION

**FIG 9:**
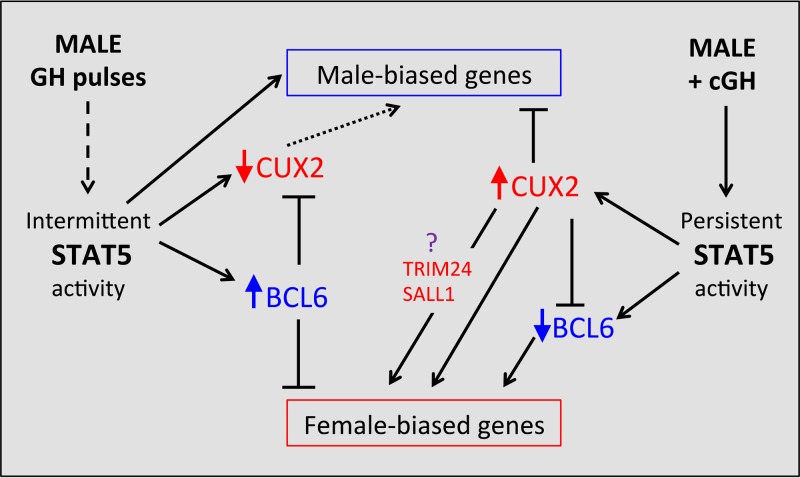
Model for role of GH-regulated transcription factors STAT5, BCL6, and CUX2 in hierarchical regulation of sex-biased genes. In untreated male liver (left), STAT5 is intermittently activated by plasma GH pulses, which enables STAT5 to activate many male-biased genes in male mouse liver, including the male-biased repressor BCL6. BCL6 represses many female-biased genes, including the female-specific repressor CUX2. *Cux2* expression is derepressed in STAT5-deficient male liver, indicating that GH pulse-activated STAT5 has a role (either direct or indirect) in the repression of CUX2 expression in male liver. The absence of CUX2 in male liver is permissive for expression of many male-biased genes (dashed arrow). cGH infusion abolishes the intermittent activity of STAT5, which leads to early downregulation of BCL6, followed by early derepression of many female-biased targets of BCL6, including *Cux2*. The expression of CUX2 in cGH-treated male liver, in turn, represses many male-biased genes and both directly and indirectly induces other female-biased genes. The indirect stimulatory effects of CUX2 on many female-biased genes are proposed to be mediated by the transcription factors TRIM24 and/or SALL1, which are both female-biased targets of CUX2. See the text for further discussion and supporting references.

**FIG 10:**
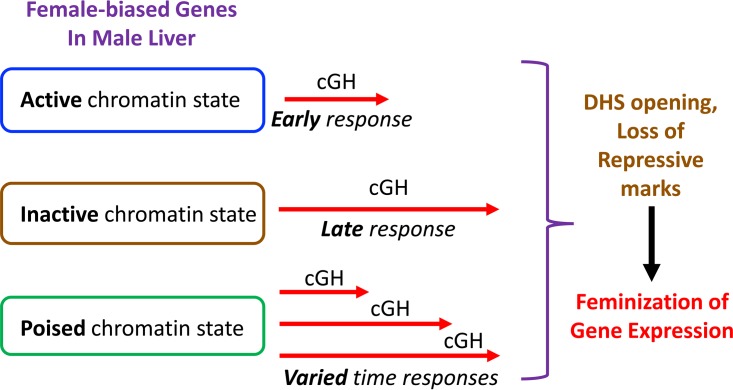
Model for the impact of chromatin state in male liver on time responsiveness of female-biased genes to cGH infusion. Female-biased genes that are already in an active chromatin state in male liver are enriched for early responses to cGH (genes in clusters F1 and F2), whereas genes in an inactive state in male liver are enriched for late responses to cGH (cluster F5 genes). Female-biased genes in a poised chromatin state in male liver showed varied cGH time responsiveness (Fig. 5B); these genes are most commonly in an active chromatin state in female liver and are therefore presumed to transition from a poised to an active chromatin state in response to cGH infusion. cGH induction of female-biased genes is proposed to involve opening of distal DHS and loss of repressive chromatin marks, as shown for several individual female-biased genes in Fig. 6 and 7. cGH induction likely also involves acquisition of activating marks. cGH repression of male-biased genes is proposed to involve DHS closing, as shown for *Gstp1* in Fig. 7, and likely also loss of activating chromatin marks.

cGH infusion overrides the male, pulsatile pattern of plasma GH and feminizes liver gene expression, with female-biased genes induced and male-biased genes repressed, as seen in rats (49, 50) and for individual genes in mice (24). Differences in the time responsiveness to cGH were reported for a small number of sex-biased genes (24, 51), but a global analysis was not previously carried out, and the mechanisms responsible for the temporal differences in responsiveness were poorly understood. Here, we used the cGH infusion mouse liver model to identify and characterize early, intermediate, and late cGH-responding sex-biased genes. Early cGH responding genes identified include several GH-regulated transcription factors that enforce liver sex differences, most notably the female-specific repressor CUX2 (33, 34, 52) and the male-biased repressor BCL6 (29, 31). Sex-biased targets of STAT5, whose temporal activation profile is directly feminized by cGH treatment (29), as well as sex-biased targets of these two repressors, are enriched in the sets of early responding genes: male cluster M2 genes are enriched for STAT5 activation and for CUX2 repression, and female cluster F1 genes are enriched for BCL6 repression. STAT5, CUX2, and BCL6 are thus part of a hierarchical transcriptional network controlling cGH-induced changes in sex-biased liver gene expression (Fig. 9). Further, the time responsiveness to cGH infusion is linked to the basal chromatin environment in male liver, with female-biased genes already in an active chromatin state in male liver enriched for early cGH responses, and genes in an inactive chromatin state enriched for late cGH responses (Fig. 10). Finally, as we illustrate for select sex-biased genes, corresponding time-dependent changes in sex-dependent local chromatin accessibility and histone modifications accompany these transcriptional responses to cGH.

cGH treatment of adult male mice substantially feminizes the liver, but the time required for feminization showed an unexpectedly large variation between genes. The earliest female-biased gene responses were seen for *Cux2* and certain other genes, whose expression in cGH-treated male liver was induced within 10 h and was fully feminized within 24 h of initiating cGH infusion. Many male-biased genes were also downregulated over this time period, while others required 4 days for downregulation (Fig. 1C). In contrast, cGH induction of many other female-biased genes was delayed for times ranging from 4 to 14 days, while other female-biased genes were resistant to the feminizing effects cGH, even after 14 days (e.g., *Cyp2b13* and four *Sult2a* genes in cluster F5). In stark contrast, <1% of stringent sex-independent genes showed a response to cGH treatment.

Our studies provide insight into the mechanisms that underpin these disparate times for feminization of sex-biased genes. First, STAT5, CUX2, and BCL6 were implicated in a hierarchical transcriptional network that controls the time dependence of liver feminization (Fig. 9). The earliest event, in which liver STAT5 activity is feminized by its switch from a pulsatile to a persistent activity pattern (29), is a direct response to persistent GH stimulation, which overrides the male pulsatile plasma GH pattern and its pulsatile activation of liver nuclear STAT5 (26, 27). This loss of pulsatile STAT5 activity leads directly to the downregulation of male-biased genes whose transcription is dependent on intermittent STAT5 stimulation. These genes include *Ces2b* and *Ugt2b38*, whose transcription in adult male liver is intermittent and directly induced by each pulse of nuclear STAT5 activity (41), as well as many other male-biased genes, whose transcriptional activation by STAT5 cannot be directly induced by a single pulse of STAT5 activity (41) but nevertheless require GH pulse-activated STAT5, as evidenced by their downregulation in STAT5 knockout mice (10, 25) and following pituitary hormone ablation (41). Consistent with this proposal, 48% of male-biased mRNAs were significantly downregulated within 1 day of cGH treatment (genes in clusters M1 and M2), and 78% were >80% feminized by day 4. The precise time required to downregulate individual male-biased RNAs likely is affected by the half-life of each individual mRNA.

Secondary events in the hierarchical transcriptional network activated by cGH infusion (Fig. 9) include the downregulation of *Bcl6*, encoding a male-biased repressor of many female-biased genes (29), and transcriptional activation of C*ux2*, encoding a highly female-specific transcription factor that directly represses a large fraction (∼35%) of male-biased genes (34). The early downregulation of *Bcl6* by cGH is expected to contribute to the observed derepression of many female-biased genes, while the induction of *Cux2*, which constitutes the strongest early response to cGH in our data set (17-fold increase after 10 h), is expected to lead to repression of many male-biased genes. ChIP-seq binding studies indicate that *Cux2* is a target for repression by BCL6 (29) and that *Bcl6* is a target for repression by CUX2 (34), highlighting the potential for mutual inhibitory cross talk between these two transcriptional repressors. CUX2 also induces ∼35% of female-biased genes by an indirect mechanism, one that does not involve direct binding by CUX2 but is proposed to be mediated by the female-biased factor TRIM24 (33) (Fig. 9). *Trim24* itself is a direct target of CUX2 (34) and was induced by cGH infusion soon after *Cux2*. CUX2 also directly binds to and induces certain highly female-biased genes (34), which we show here are significantly enriched in the late cGH-responding gene cluster F5. *Cux2* induction (which occurs early) is thus not a determinant of the unusually slow feminization of cluster F5 genes. Taken together, these findings support a multistep hierarchical network, whereby early repression of a male-biased repressor of female-biased genes (*Bcl6*) and early induction of a female-biased activator of female-biased genes and repressor of male-biased genes (*Cux2*) contribute to the feminization of many downstream cGH-responsive sex-biased genes.

We identified SALL1 as a female-biased transcriptional regulator (Table 2) and CUX2 target (34) that may also contribute to transcriptional responses downstream of CUX2 (Fig. 9). Notably, SALL1 is a transcriptional repressor that interacts with the nucleosome remodeling and deacetylase complex NuRD (53) and could contribute to CUX2 repression of male-biased genes in female liver. Similar to *Trim24*, *Sall1* is a direct target of CUX2 binding, is downregulated following *Cux2* knockdown in female liver (34), and acquires its female specificity in mouse liver at 4 weeks postnatal age, i.e., prior to most sex-biased genes (54). Further study is needed to determine the precise role of SALL1 in the transcriptional hierarchy controlling sex-biased liver gene expression.

A second, complementary mechanism that impacts the time course for cGH feminization of male liver relates to the sex-specific effects of GH on liver chromatin states. The liver epigenome is characterized by extensive GH-dependent sex-differences in open chromatin regions (DHS) (36) and their chromatin states, as well as sex differences in local chromatin marks at a subset of sex-biased genes (37). We found that female-biased genes already in an active chromatin state tend to be more rapidly induced by cGH than genes whose induction requires extensive chromatin remodeling (Fig. 10). Examples of the changes in chromatin states induced by cGH infusion were provided by our examination of changes in sex-biased DHS and repressive chromatin marks associated with several highly sex-biased genes. Thus, cGH-induced a time-dependent loss of male-biased open chromatin proximal to the male-biased *Gstp1* that parallels its repression, and cGH induced chromatin opening and a loss of a key repressive mark (H3-K27me3) with the induction of several female-biased genes. The mechanism whereby these epigenetic changes are induced by the switch from a male to female plasma GH profile is unknown but is expected to be complex. Factors likely contributing to the changes in chromatin accessibility and chromatin states induced by cGH infusion include hormone-responsive chromatin remodeling enzymes (55, 56) and GH-regulated epigenetic regulators, including sex-biased long noncoding RNAs (57), working in combination with the GH-responsive transcriptional activators and repressors discussed above.

While a large majority of sex-biased genes were fully feminized by cGH infusion, many genes were only partially feminized, even after 14 days of cGH treatment. Examples include several highly female-specific *Sult* and *Cyp* genes (Fig. 1A). In contrast, cGH infusion of hypophysectomized or pituitary GH secretion-deficient (*little*) female mice is sufficient to fully recapitulate female-biased expression of some of the same sulfotransferase genes (*Sult2a1* and *Sult2a2*) (58). The partial feminization seen here, where cGH was infused in intact male mice, suggests intact male liver may be intrinsically less responsive to cGH than intact female mouse liver. Consistent with this proposal, male and female livers show intrinsic differences in responsiveness when plasma GH pulses are given to hypophysectomized mice (22), and primary female rat hepatocyte cultures show greater intrinsic responsiveness to cGH than male hepatocytes (59). In the rat model, pulsatile GH exposure of male liver during the early postnatal period apparently programs the male liver for GH pulse responsiveness later in life, and this is required for full adult expression of sex-biased genes (60). Accordingly, cGH infusion in adult male mice, as in the present study, may not be sufficient to abolish the imprinting effects of neonatal GH pulse exposure, and consequently, cGH exposure of adult males may not fully reverse the male chromatin state. Supporting this idea, male mice with a knockout of *Somatostatin*, which feminizes plasma GH profiles by abolishing the hormone-free trough period between plasma GH pulses, induces *Sult* genes to the level of the intact female (19). Presumably, *Somatostatin* deficiency during early male development is sufficient to abolish any programming effect of neonatal GH pulse exposure, and thus eliminates any intrinsic sex difference in the liver's responsiveness to the female plasma GH profile. Further study is needed to determine the role of these, presumably epigenetic mechanisms in cGH responses.

GH regulates gene expression primarily at the level of transcription initiation, as evidenced by run-on transcription assays and by the analysis of hnRNA for several individual genes (41, 61). Our global analysis of cGH-responsive genes using RNA-seq intronic reads provides further evidence for transcriptional regulation of liver sex differences by GH, since the nascent transcripts represented by these intronic reads (40, 62) showed cGH response patterns indistinguishable in many cases from those of mature mRNAs. Two early cGH-responding male-biased genes, *Ces2b* and *Ugt2b38*, showed significantly greater early downregulation responses at the intronic read (primary transcript) level (Fig. 3C). Transcription of these genes responds rapidly and dynamically to endogenous GH pulses (41), and consequently, these male-biased genes are expected to be uniquely sensitive to the loss of pulsatile STAT5 activation secondary to cGH infusion.

Hepatocytes near the portal vein show substantial differences in metabolic and other functions compared to hepatocytes near the central vein (45, 46) and may be exposed to different concentrations of GH (45). Indeed, many *Cyp* genes show a pericentral bias in expression that can be regulated by GH, with liver zonation abolished for a subset of rat *Cyp* genes by hypophysectomy and restored by cGH infusion (45, 63). We used liver zonation profiles recently reported (48) to examine whether genes showing a zonated pattern of expression show different kinetics of response to cGH or show a difference in sensitivity to a change in circulating GH that may account for the partial feminization of some sex-biased genes by cGH. However, although half of the 229 sex-biased genes we examined showed significant differential zonated distribution of expression between periportal and pericentral hepatocytes (Fig. 8), we saw no significant differences in zonation bias between early and late cGH-responding genes.

The present model of cGH infusion in adult male mice uses a cGH dose and regimen that produces normal female physiological plasma GH levels in a rat model (66) and overrides the male, pulsatile pattern of mouse liver STAT5 activation (29). The persistence of plasma GH achieved in these studies is markedly different from the pathological states of elevated GH exposure, where Igf1 production shows major dysregulation in both GH resistance (IGF1 suppression) and acromegaly (IGF1 induction) (67). In contrast, cGH treatment did not significantly alter liver *Igf1* expression, consistent with the similar levels of liver IGF1 production between the sexes and with cGH infusion being a physiological and not a pathophysiological model.

In conclusion, the time-dependent patterns of repression of male-biased genes and derepression of female biased genes by cGH infusion are dictated by hierarchical transcriptional networks and by the dynamics of the local sex-biased epigenetic states. These networks and their associated epigenetic control mechanisms are likely to impact the widely observed sex differences in liver metabolism and disease susceptibility, including liver cancer. Indeed, cGH infusion alters the expression of several genes involved in hepatocellular carcinoma pathogenesis. These include *Trim24*, a female-biased transcriptional regulator and tumor suppressor for hepatocellular carcinoma (64) that is induced by cGH within 10 h, and *Nox4*, a cGH-repressed male-biased gene involved in liver oxidative stress and stellate cell activation (65). Further studies are required to elucidate the cGH-responsive distal regulatory elements and their interactions with proximal promoters of sex-biased genes.

## MATERIALS AND METHODS

### Animal treatments.

Male and female CD1 mice [Crl:CD(ICR); strain code 022], 8 to 9 weeks of age, were purchased from Charles River Laboratories (Kingston, NY). Mice were housed in a temperature and humidity controlled environment with a 12-h light, 12-h dark cycle, fed standard rodent chow, and supplied with tap water. Alzet osmotic pumps (model 1007D; Durect Corp., Cupertino, CA) were filled with recombinant rat GH (purchased from Arieh Gertler, Protein Laboratories Rehovot, Ltd., Rehovot, Israel) dissolved in 30 mM NaHCO_3_ and 0.15 M NaCl containing 0.1 mg/ml rat albumin (Sigma-Aldrich, catalog no. A6414). Pumps were implanted subcutaneously under ketamine and xylazine anesthesia. GH was infused at a rate of 25 ng/g (body weight)/h for periods ranging from 10 h to 14 days. To minimize the impact of stress on early changes in gene expression caused by pump implantation, initiation of GH delivery for the 10-h to 7-day time points was delayed for a period of 96 h. This delay was achieved, as suggested by the manufacturer, by attaching to each pump a coiled polyethylene catheter (PE-60, catalog no. 0007750, lot PE00008; Durect Corporation, Cupertino, CA) filled with saline buffer whose length and flow rate correspond to the 96-h delay period, as follows. The measured rate of saline flow through a coiled catheter was used to calculate a catheter length (0.99 mm/h × 96 h). An extra 4-mm length was added to the calculated catheter length to adjust for the 4-mm length required to attach the catheter to the pump's flow moderator. This 96-h delay was not introduced for the 14-day GH infusion time point, since preliminary studies showed that stress-induced gene expression changes (e.g., induction of acute phase genes *Cxcl1* and *Saa3*) (68) dissipated within a few days. However, to ensure a full 14-day exposure to bioactive GH, Alzet pumps were removed 7 days after initiating cGH infusion (i.e., 96 h plus 7 days after pump implantation) and replaced with a new Alzet minipump delivering fresh GH for an additional 7 days. Untreated female and sham-treated male mice (i.e., Alzet pumps filled with buffer) were used as controls. Mice were euthanized by cervical dislocation at a consistent time of day (9 a.m.). All animal procedures were approved by the Boston University Institutional Animal Care and Use Committee.

### Nuclear extraction and chromatin cross-linking.

Fresh liver tissue was excised from individual mice and homogenized on ice in a Potter-Elvehjem tissue grinder using 12 ml of homogenization buffer (10 mM HEPES [pH 7.6], 25 mM KCl, 0.15 mM spermine, 0.5 mM spermidine, 1 mM sodium orthovanadate, 10 mM NaF, 1 mM EDTA, 2 M sucrose, and 10% glycerol) with complete protease inhibitor cocktail (1 tablet/25 ml; Sigma-Aldrich, catalog no. 11697498001). The homogenate was loaded onto a 3-ml cushion of homogenization buffer on Ultra-Clear ultracentrifugation tubes (Beckman, catalog no. 344059) and centrifuged in a Sorvall Pro ultracentrifuge in a SW41 Ti rotor at 4°C at 25,000 rpm for 30 min. The pelleted nuclei obtained from approximately 30 to 35% of the liver homogenate were resuspended in 400 μl of nuclei storage buffer (20 mM Tris-HCl [pH 8.0], 75 mM NaCl, 0.5 mM EDTA, 50% glycerol, 0.85 mM dithiothreitol, 0.125 mM phenylmethylsulfonyl fluoride) using a 0.5-ml Dounce homogenizer. Samples were snap-frozen and stored at −80°C until further use for DNase hypersensitivity analysis. The pelleted nuclei from the remaining two thirds of each liver were resuspended in 1 ml of cross-linking buffer (10 mM HEPES [pH 7.6], 25 mM KCl, 0.34 M sucrose, 0.15 mM 2-mercaptoethanol, 2 mM MgCl_2_) and incubated with 0.8% formaldehyde (final concentration) for 9 min in a 30°C water bath with periodic shaking. Cross-linking was halted by addition of a 1 M glycine (pH 8.0) solution (final concentration, 0.1 M), followed by incubation for 5 min at room temperature. Samples were placed on ice, layered on a 3-ml cushion of homogenization buffer, and centrifuged at 4°C at 25,000 rpm for 30 min. The cross-linked nuclear pellet was resuspended in 1 ml of 1× radioimmunoprecipitation assay buffer (50 mM Tris Cl [pH 8.1], 150 mM NaCl, 1% IGEPAL CA-630 [Sigma-Aldrich, catalog no. I8896], 0.5% sodium deoxycholate) containing 0.5% sodium dodecyl sulfate and Complete protease inhibitor cocktail. Samples were sonicated in borosilicate glass tubes (Rim Top, N-51A; Kimble Chase, catalog no. 73500-1275) using a Branson 250D Sonifier with a double-stepped microtip (35% amplitude for 15 s “ON” and 45 s “OFF” for 30 cycles) or using a Diagenode Bioruptor UCD-400 Twin in 15-ml polystyrene tubes (BD Falcon Cat 352095) for 75 to 80 cycles (30 s ON and 30 s OFF, high intensity). A 15-μl aliquot of the sonicated chromatin was reverse cross-linked, treated with RNase A and proteinase K, and electrophoresed on a 1% agarose gel to size the fragments. For more details, see reference 29. The majority of DNA fragments ranged from 100 to 300 bp. The remaining sonicated chromatin was snap-frozen in liquid nitrogen and stored at −80°C until further use for ChIP.

### ChIP and DNase hypersensitivity assay.

Chromatin immunoprecipitation (ChIP) was performed as reported previously (29) using the following ChIP-validated antibodies: H3K27me3 (Abcam, catalog no. ab6002; 2 μg of antibody per 10 μg of sonicated chromatin) and normal rabbit IgG (Santa Cruz, catalog no. sc-2027; 3 μg of antibody per 15 μg of sonicated chromatin). ChIP DNA was quantified using a Quant-iT PicoGreen assay kit (Invitrogen) and analyzed by qPCR to interrogate genomic regions selected as positive controls or as negative controls for H3K27me3, generally based on published ChIP-seq data (37). DNase I digestion of liver nuclei (36) was performed as previously described (41). Briefly, 25 × 10^6^ liver nuclei per sample were incubated with 32 U of RQ1 RNase-free DNase I (1 U/μl; Promega) for 2 min at 37°C. Samples were extracted with phenol-chloroform and then loaded on a sucrose gradient and centrifuged at 25,000 rpm for 24 h at 25°C. Individual fractions were collected and analyzed on a SYBR green-stained agarose gel to determine the fragment size distribution. Fractions enriched in DNA fragments between 100 bp and 1 kb in size (median size, 300 bp) were pooled. Fragments 125 to 400 bp in length were isolated using a dual SPRI size selection with Agencourt AMPure XP beads (Beckman Coulter, catalog no. A63881). A SPRI/DNA ratio of 0.6 was used to remove fragments of >400 bp, and a SPRI/DNA ratio of 1.9 was used to retain fragments of >125 bp. DNA was quantified using a Quant-iT PicoGreen assay kit (Invitrogen) and qualified by qPCR using primers that amplify genomic sequences previously identified as liver DHS (36).

### RNA isolation and sequencing.

Approximately 10% of each individual liver was snap-frozen in liquid nitrogen and used for RNA extraction with TRIzol reagent (Invitrogen Life Technologies, Inc., Carlsbad, CA) according to the manufacturer's instructions. Total liver RNA was isolated from livers of cGH-infused or sham-treated male mice. Two or three sequencing libraries (biological replicates) were prepared for each time point of cGH treatment (i.e., 10 h, 1 day, 4 days, 7 days, and 14 days of GH infusion). For the 1-, 4-, and 7-day cGH time points, each sequencing library was prepared from a single mouse liver. For the 14-day time point and for the sham-treated males, each sequencing library corresponded to a pool of *n* = 2 or 3 individuals. We observed high individual variability in gene responses after 10 h of GH infusion; therefore, we expanded the number of individual mouse livers at that time point to give a pool of *n* = 9 or 10 individual livers for each of two sequencing libraries. Sequencing libraries were prepared using the Illumina TruSeq RNA library preparation kit (Illumina, catalog no. RS-122-2001). Total liver RNA was also isolated from a large number of control (untreated) male and female mouse livers, from which we prepared three RNA pools per sex, each comprised of RNA from *n* = 12 to 17 individual livers. In addition, total RNA isolated from livers of sham-treated males, control females, and from mice given cGH infusion for 10 h or 1 day were used to prepare three pools per condition, using *n* = 2 to 4 livers per pool. The latter RNA samples were treated with DNase and then depleted of rRNA using the NEBNext rRNA depletion kit (NEB, catalog no. E6310). Sequencing libraries were prepared using NEBNext Ultra Directional RNA Library Prep kit for Illumina (NEB, catalog no. E7420) according to the manufacturer's instructions. NEBNext multiplex oligonucleotides for Illumina (NEB, catalog no. E7335) were used for multiplexing. The Agencourt AMPure XP system (Beckman Coulter, catalog no. A63880) was used for sample and library purification. Library quality and size distribution were assessed using an Agilent Bioanalyzer DNA high-sensitivity kit (Agilent Technologies, catalog no. 5067-4627). Sequencing on an Illumina HiSeq2000 or HiSeq2500 instrument was carried out at the New York Genome Center (New York, NY) or at the Boston University Department of Computational Biomedicine (Boston, MA), generating 50-, 100-, or 125-nucleotide paired-end sequence reads.

### RNA-seq and identification of sex-biased and GH-responsive genes.

RNA-seq data were analyzed using a custom pipeline developed in our lab and recently described (41). Briefly, sequence reads were aligned to mouse genome build mm9 (NCBI 37) using Tophat (version 2.0.13) (69), and FeatureCounts (70) was used to count sequence reads mapping to three different sets of genomic regions: collapsed exon (the union of the exonic regions in all isoforms of a given gene), exonic only (exonic regions excluding those exonic sequences that overlap an intron in any of the isoforms), and intronic only (the union of the intronic regions in all isoforms of a given gene, excluding those intronic sequences that overlap an exon in any of the isoforms). Differential expression analysis was conducted using the Bioconductor package EdgeR (71). We identified 9,709 liver-expressed genes based on fragments per kilobase length of transcript per million mapped reads (FPKM) values of >1 in either male or female liver. A total of 983 sex-biased genes were identified based on thresholds of an EdgeR adjusted *P* value of < 0.01 and an FPKM of >1, which empirically corresponded to a >1.2-fold sex-difference in expression (see the gene listing in Table S1 in the supplemental material).

A stringent set of 255 sex-biased genes was defined based on an adjusted *P* value of <0.01 and showing a >2-fold sex-difference when comparing adult male to adult female liver; this list excludes four Y-chromosome genes (see Tables S2 and S3 in the supplemental material). These genes also show a >2-fold ratio when comparing the FPKM values of the sham-treated males to those of control females. For each sex-biased gene, a percent feminization value was calculated based on responses to cGH infusion, as follows: % feminization = 100% × [FPKM (cGH-infused male) − FPKM (sham-treated male)]/[FPKM (control female) − FPKM (sham-treated male)]. A gene was considered responsive to cGH infusion if its differential expression analysis met the threshold of adjusted *P* value of <0.05 and if the gene showed ≥20% feminization at a given time point. A set of 7,225 stringent sex-independent genes was defined based on an EdgeR adjusted *P* value of >0.1 and a fold change for sex difference of <1.2. Only 62 of these 7,225 sex-independent genes showed a response to cGH at one or more time points, and only 14 of the 62 genes were consistently induced or consistently repressed by cGH and showed >4-fold maximal response, whereas 11 genes showed significant responses only after 14 days of cGH infusion (see Tables S4 and S5 in the supplemental material).

### Clustering of cGH-responsive genes.

STEM (Short Time-series Expression Miner) (38, 72) was used for initial analysis of the patterns of response to cGH infusion. STEM defines a set of model response profiles independent of the data. Each gene was assigned to the predefined STEM profile that most closely resembled its time-dependent response to cGH infusion, and STEM profiles with a minimum correlation value were clustered together. STEM clustering of the set of 255 sex-biased genes (EdgeR, adjusted *P* value [*P*_adj_] < 0.01, fold change > 2; see Tables S2 and S3 in the supplemental material) was carried out using the percent feminization values (described above) for each gene at each cGH treatment time point. The maximum number of model STEM profiles was set to 8, the maximum unit change in model profiles between time points was set to 10, the minimum correlation to cluster profiles was set to 0.7, and significance values represent the false discovery rate (FDR). After excluding 22 unresponsive genes (genes that did not meet the EdgeR *P* value threshold), STEM analysis revealed that 198 of the 233 cGH-responsive sex-biased genes (85%) showed a time-dependent increase in feminization represented by STEM profile 4 (FDR 7.0E−147). These 198 genes were comprised of male-biased and female-biased genes with a progressive, time-dependent pattern of feminization (see Tables S2 and S3 in the supplemental material). Sixteen other cGH-responsive genes with the following properties were absent from STEM profile 4. These 16 genes were all female-biased but either responded to cGH only after 14 days or were highly induced by cGH; nevertheless, the maximal extent of feminization based on FPKM values was <20%. For example, *Sult2a2* was induced by cGH 919-fold after 14 days, to an FPKM of 9.73, which corresponds to 12.8% of the female FPKM of 76.3 (see Table S1 in the supplemental material). These two gene lists (i.e., the 198 + 16 = 214 sex-biased genes) were further clustered based on the cGH time course, as follows. Heat map generation and hierarchical clustering were conducted using Morpheus (https://software.broadinstitute.org/morpheus/). Complete-linkage hierarchical clustering of the feminization values at each time point was implemented, and led to our identification of five clusters of cGH-responsive male-biased genes, designated M1 to M5, and five clusters of cGH-responsive female-biased genes, designated F1 to F5. One gene (*Igsf23*) was filtered out by this clustering, and the remaining 213 clustered sex-biased genes were used for all downstream analyses. Functional annotation analysis was performed using DAVID (73).

### Mapping binding sites to genes and enrichment for sex-biased gene targets.

Male-enriched, female-enriched and sex-independent STAT5 binding sites (1,765, 1,790, and 11,531 sites, respectively [as defined in reference 29]), BCL6 binding sites in male liver (6,432 sites), and CUX2 binding sites in female liver (1,471 sites) were obtained from our published ChIP-seq data (29, 34) (GEO accession numbers GSE31578 and GSE35985). Male-biased (*n* = 2,800), female-biased (*n* = 1,379), and sex-independent (*n* = 68,682) DHS were also obtained from our published data (36) (GEO accession number GSE21777). Each set of regulatory elements was mapped to its putative gene targets using GREAT (Genomic Regions Enrichment of Annotations Tool [43]) according to the following parameters. Each RefSeq gene was assigned a basal regulatory domain extending from 5 kb upstream to 1 kb downstream of the TSS, and the regulatory domain was extended in both directions to the nearest gene's basal regulatory domain up to a maximum of 1,000 kb in one direction (43). Enrichment results obtained using this mapping method were similar to those obtained when transcription factor binding sites were mapped to the nearest gene within 10 kb, as was done previously (29, 34; also, data not shown). For STAT5 and for DHS, the gene targets of male-enriched sites were filtered to remove any genes that were also identified as a target of a female-enriched site and correspondingly for the gene targets of female-enriched sites. Gene targets of sex-independent STAT5 binding sites or DHS were defined as those identified by GREAT as a target of one or more sex-independent sites but that did not map to any male-enriched or female-enriched transcription factor binding site or DHS. The enrichment of sex-biased genes in each set of transcription factor targets was calculated compared to that of a background set of stringent sex-independent genes (see Table S5 in the supplemental material). The enrichment of the transcription factor targets for belonging to each of the 5 cGH response clusters in each sex (designated M1 to M5 and F1 to F5; see Table 1 and below) was calculated compared to the set of corresponding GH-responsive sex-biased genes not in that cluster. Statistical significance was evaluated by using the Fisher exact test.

### Enrichment for liver chromatin states.

We previously clustered all liver-expressed genes by the read densities of seven chromatin features across a 2-kb window centered at the TSS and a 2-kb window centered at the TES (37). Clusters of genes were defined as either active (clusters 1, 2, and 3 of Fig. S5 in reference 37), poised (clusters 4 and 5 of Fig. S5 in reference 37), or inactive (cluster 6 of Fig. S5 in reference 37) based on the relative density of DNase hypersensitivity, activating chromatin marks (H3-K27ac, H3-K4me1, H3-K4me3, and H3-K36me3) and repressive chromatin marks (H3-K27me3 and H3-K9me3). A total of 158 of the 213 sex-biased genes grouped into clusters M1 to M5 and F1 to F5, as described above, were available for analysis of enrichment for liver chromatin states after removal of 38 genes <5 kb in length (to exclude genes whose TSS-proximal sequences overlap the TES-associated region) and after removing 17 genes for which the earlier chromatin state analysis (37) was not performed. Genes from this list of 158 genes that were found in each of the five cGH response clusters in each sex were tested for their being enriched in active, poised, or inactive chromatin states compared to the set of corresponding cGH-responsive sex-biased genes not in that cluster. The statistical significance was calculated using the Fisher exact test. Clustering and enrichments were carried out separately in male and female livers. For example, 29 of the 53 female-biased genes in clusters F1 and F2 (see Table S3 in the supplemental material) are in an active chromatin state in the male liver compared to 10 of 46 genes in the other clusters (F3 to F5). An enrichment score of 4.35 (Fig. 5B and below) was calculated as follows: [29 (F1+F2) genes in active state/10 (F3, F4, and F5) genes in active state]/[24 (F1+F2) genes not in active state]/[36 (F3, F4, and F5) genes not in active state]. A significant *P* value of 0.00098 was obtained by using the Fisher exact test.

### qPCR analysis.

Liver total RNA (1 μg) was used for reverse transcription (RT) using the Applied Biosystems high-capacity cDNA reverse transcription kit (Fisher, catalog no. 43-688-14). qPCR was performed using Power SYBR green PCR master mix and processed on an ABS 7900HT sequence detection system (Applied Biosystems) or the CFX384 Touch real-time PCR detection system (Bio-Rad). For RT-qPCR, raw *C_T_* values were analyzed using the comparative *C_T_* method with normalization to the 18S RNA content in each sample. Raw *C_T_* values for DHS samples were normalized to the DHS level in each sample at a prominent, sex-independent DHS near *Tram2* (mm9 genomic coordinates: chr1, 21049167 to 21050218). Raw *C_T_* values for H3-K27me3 ChIP-qPCR were normalized to the H3-K27me3 level in the *Hoxb* gene region (mm9 genomic coordinates: chr11, 96135000 to 96145000, 2.1 kb upstream of *Hoxb8*). qPCR primers were designed using Primer Express software (Thermo Fisher Scientific, Inc.). Primers were specific for their target sequences as verified using the BLAT function of the UCSC browser after extending the sequence to include 3 to 10 nucleotides of flanking sequence on each end, as described previously (41). Primary RNA transcripts (hnRNA) were quantified using a primer pair spanning an exon-intron junction. Primers used to quantify mature (spliced) mRNAs were targeted to adjacent exons separated by an intron >1 kb in length (with the exception of two primer pairs that were <1 kb apart) to minimize the likelihood of having contaminating genomic DNA contribute to the qPCR signal. Primer sequences are shown in Table S6 in the supplemental material.

### Analysis of liver zonation of cGH-responsive sex-biased genes.

We used liver zonation data reported in a recent study (48) that combined single-molecule fluorescence *in situ* hybridization and single-cell RNA-seq data to establish the zonation profiles of all hepatocyte-expressed genes in adult male liver. Zonation layers in the liver lobule were numbered from zone 1 (closest to central vein) to zone 9 (most periportal), and the expression values in each zone were determined for every liver expressed gene. We obtained the zonation profiles for 229 sex-biased genes and for 6,672 stringent sex-independent genes. Genes were defined as being significantly zonated if they met a *q* value of <0.2 for differential zonation as described previously (48). For each of the significantly zonated genes, a zonation bias ratio was calculated as follows: zonation bias = [expression in zone 1 (closest to central vein) − expression in zone 9 (most periportal)/(mean expression in all nine zones)]. Zonation ratios were compared for sex-biased genes versus sex-independent genes and for clusters 1 and 2 (early) versus clusters 3 to 5 (not-early) cGH-responsive female-biased genes and, separately, male-biased genes. *P* values were obtained by Wilcoxon rank sum tests.

### Statistical analysis.

Graphical and statistical analyses were performed using GraphPad Prism 7 software. RNA, DHS, and ChIP qPCR data are expressed as mean values ± the standard errors of the mean (SEM) for *n* = 6 to 10 individual mouse livers per group, as specified in each figure legend. One-way analysis of variance (ANOVA) with a Dunnett posttest was used to compare each treatment group to its sham-treated control group as noted in the appropriate figure legends. Statistical significance reached by a *t* test but not ANOVA is indicated in the appropriate figure legends. Statistical significance of all the enrichment or depletion calculations was evaluated by using the Fisher exact test.

### Accession number(s).

All raw and processed RNA-seq data are available under accession number GSE98586 at Gene Expression Omnibus (https://www.ncbi.nlm.nih.gov/gds/).

## Supplementary Material

Supplemental material
