# Supplementary material for: Feminization of Male Mouse Liver by Persistent Growth Hormone Stimulation: Activation of Sex-Biased Transcriptional Networks and Dynamic Changes in Chromatin States
Source: Mol Cell Biol. 2017 Sep 12;37(19):e00301-17. doi: 10.1128/MCB.00301-17 (PMC5599723; doi:10.1128/MCB.00301-17)
Supplement: Supplemental material [file supp_37_19_e00301-17__index.html]

Feminization of Male Mouse Liver by Persistent Growth Hormone Stimulation: Activation of Sex-Biased Transcriptional Networks and Dynamic Changes in Chromatin States — Supplemental material 

# Feminization of Male Mouse Liver by Persistent Growth Hormone Stimulation: Activation of Sex-Biased Transcriptional Networks and Dynamic Changes in Chromatin States

## Supplemental material

- Supplemental file 1 -

  Fig. S1 (Analysis of intronic sequence reads from polyA+ selected RNA-seq) and S2 (H3K27me3 abundance on female-biased genes)

  PDF, 332K
- Supplemental file 2 -

  Tables S1 (Differential expression analysis for 983 liver sex-biased genes), S2 and S3 (Characterization of 113 male-biased [S2] and 142 female-biased [S3] genes), S4 (Differential expression analysis for 62 stringent sex-independent genes), S5 (Transcription factor binding sites and DNase hypersensitive sites associated with 7,225 stringent sex-independent genes), S6 (Primer sequences for qPCR), S7 (KEGG pathway analysis), and S8 (Differential expression analysis for 226 sex-biased genes)

  XLSX, 1.5M
