# Supplementary material for: Feminization of Male Mouse Liver by Persistent Growth Hormone Stimulation: Activation of Sex-Biased Transcriptional Networks and Dynamic Changes in Chromatin States
Source: Mol Cell Biol. 2017 Sep 12;37(19):e00301-17. doi: 10.1128/MCB.00301-17 (PMC5599723; doi:10.1128/MCB.00301-17)

Supplemental Figures and Tables

Feminization of male mouse liver by persistent growth hormone stimulation: Activation of sex-biased transcriptional networks and dynamic changes in chromatin states

Dana Lau-Corona, Alexander Suvorov and David J. Waxman*

All supplemental figures can be found in this pfd. Supplemental tables, in Excel format can be found as Tables S1-S8.

Supplemental Figures

Figure S1 - Analysis of intronic reads from PolyA+ selected RNA-seq
Figure S2 - H3K27me3 abundance on female-biased genes

Supplemental Tables

Table S1 - Differential expression analysis for 983 Liver Sex biased genes (FPKM >1, EdgeR Adjusted p<0.01) based on PolyA+ RNA-seq Exon collapsed counts.
Table S2 – Characterization of 113 Male-biased genes (FPKM >1, EdgeR p<0.01, FC >2) based on their response to continuous GH infusion and their transcriptional and epigenetic regulation.
Table S3 - Characterization of 142 Female-biased genes (FPKM >1, EdgeR p<0.01, FC >2) based on their response to continuous GH infusion and their transcriptional and epigenetic regulation.
Table S4 – Differential expression analysis for 62 Stringent sex-independent genes (FPKM >1, EdgeR p>0.1, FC <1.2) that are responsive to continuous GH (EdgeR p<0.05, FC >2).
Table S5 – Transcription factor binding sites and DNAse Hypersensitive sites (DHS) associated to 7,225 Stringent sex-independent genes (FPKM >1, EdgeR p>0.1, FC <1.2).
Table S6 - Primer sequences used for qPCR analysis.
Table S7 − KEGG Pathway analysis.
Table S8 - Differential expression analysis for 226 sex-biased genes with sufficient intronic read counts from rRNA-depleted RNA-seq libraries.

Supplemental Figure Legends

Figure S1. Analysis of intronic sequence reads from PolyA+ selected RNA-seq (A) Percentage of intronic reads in PolyA+ RNA-seq compared to Total (rRNA depleted) RNA-seq. Shown are the values for the same Male and Female liver samples. (B) Heatmaps showing the Log2 ratios of the expression values for the control females and the cGH treated males at each time poin, over the expression values of the sham-treated males, obtained from <u>exonic and intronic</u> read counts from PolyA+ RNA-seq for the 113 male-biased and the 142 female-biased genes.

Figure S2. H3K27me3 abundance on female-biased genes UCSC genome browser screenshots from K27me3 ChIP-seq data for male and female mouse liver (29) on the following female-biased genes (A) *Cux2*, (B) *Cyp3a16*, (C) *A1bg* and (D) *Cyp2b9*

Figure S1

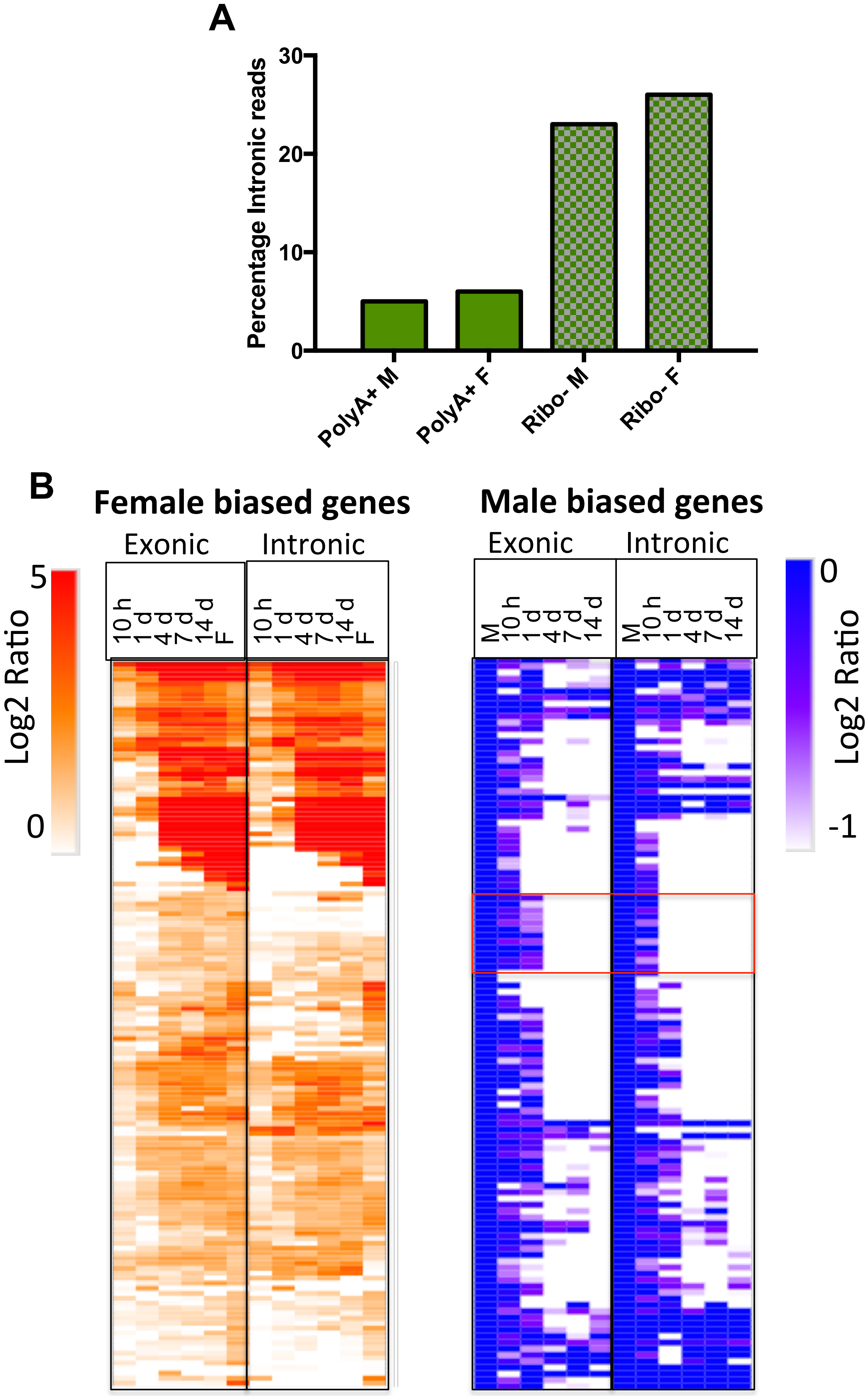
A
Percentage Intronic reads
30
20
10
0
PolyA+ M
PolyA+ F
Ribo- M
Ribo- F
B
Female biased genes
Exonic
Intronic
10 h
1 d
4 d
7 d
14 d
F
Male biased genes
M
Log2 Ratio
5
0
-1

Figure S2

A. *Cux2*

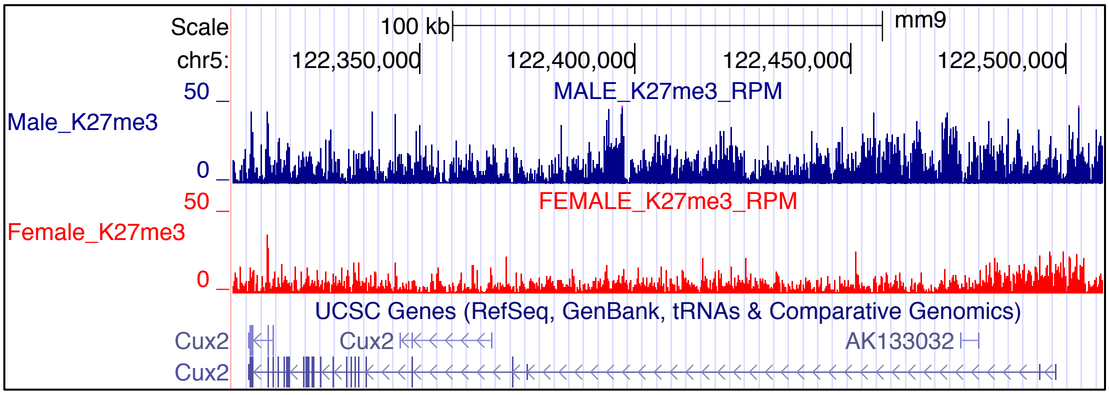

B. *Cyp3a16*

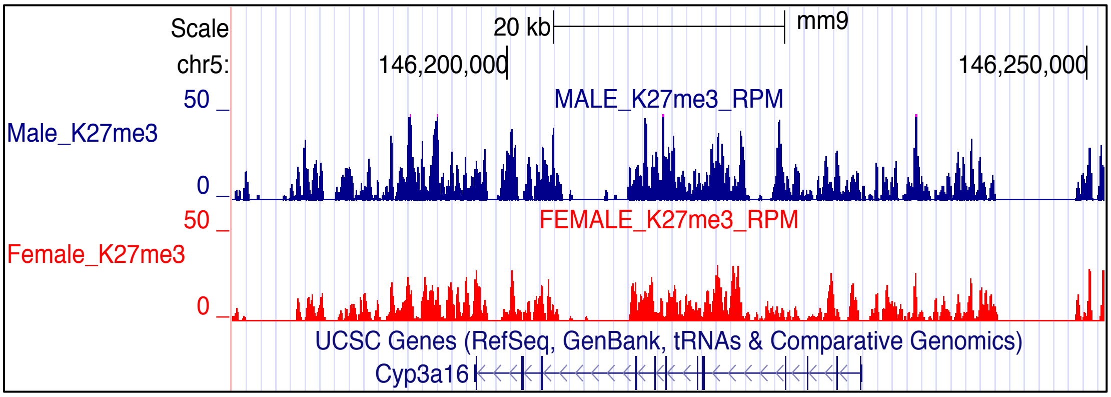

C. *A1bg*

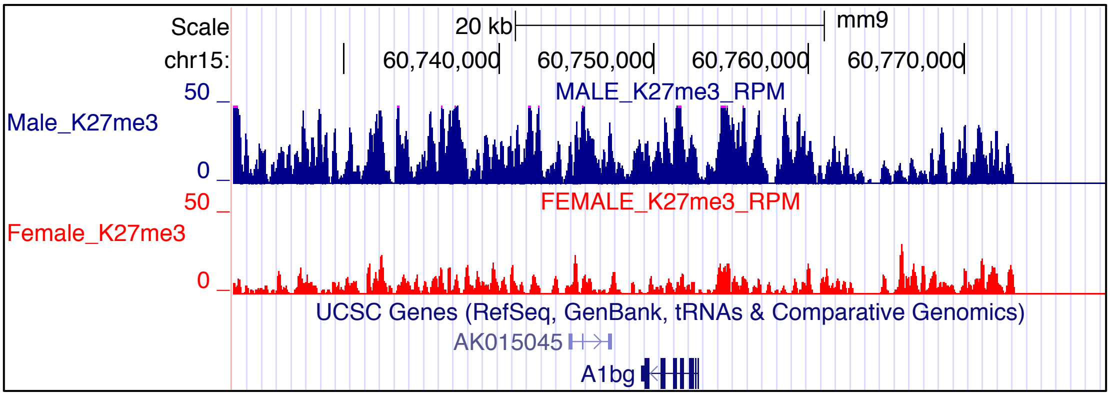

D. *Cyp2b9*

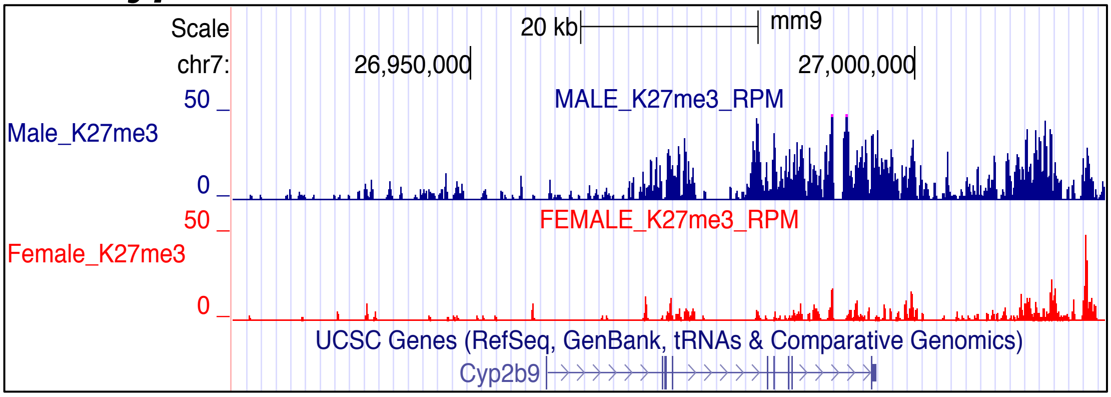

Supplement: Supplemental material [file MCB.00301-17_zmb999101604s1.pdf]
